# Research of TPU Materials for 3D Printing Aiming at Non-Pneumatic Tires by FDM Method

**DOI:** 10.3390/polym12112492

**Published:** 2020-10-27

**Authors:** Jun Wang, Bin Yang, Xiang Lin, Lei Gao, Tao Liu, Yonglai Lu, Runguo Wang

**Affiliations:** 1 Key Laboratory of Beijing City for Preparation and Processing of Novel Polymer Materials, Beijing University of Chemical Technology, Beijing 100029, China; wangjun07292020@163.com (J.W.); yb2019210196@163.com (B.Y.); luyonglai@mail.buct.edu.cn (Y.L.); 2 School of Chemistry and Biological Engineering, University of Science and Technology Beijing, Beijing 100083, China; xiang003.buct@163.com; 3 State Key Laboratory of Automotive Simulation and Control, Jilin University, Changchun 130022, China; 15754306037@163.com (L.G.); taoliu19@mails.jiu.edu.cn (T.L.)

**Keywords:** thermoplastic polyurethanes (TPU), fused deposition modeling (FDM), 3D printing process

## Abstract

3D printing technology has been widely used in various fields, such as biomedicine, clothing design, and aerospace, due to its personalized customization, rapid prototyping of complex structures, and low cost. However, the application of 3D printing technology in the field of non-pneumatic tires has not been systematically studied. In this study, we evaluated the application of potential thermoplastic polyurethanes (TPU) materials based on FDM technology in the field of non-pneumatic tires. First, the printing process of TPU material based on fused deposition modeling (FDM) technology was studied through tensile testing and SEM observation. The results show that the optimal 3D printing temperature of the selected TPU material is 210 °C. FDM technology was successfully applied to 3D printed non-pneumatic tires based on TPU material. The study showed that the three-dimensional stiffness of 3D printed non-pneumatic tires is basically 50% of that obtained by simulation. To guarantee the prediction of the performance of 3D printed non-pneumatic tires, we suggest that the performance of these materials should be moderately reduced during the structural design for performance simulation.

## 1. Introduction

Thermoplastic polyurethanes (TPU) are linear segmented block polymers that are prone to microphase separation due to the thermodynamic incompatibility between polar hard segments and relatively nonpolar soft segments [1,2,3]. The hard segments (HS) of TPU are typically derived from diisocyanates and small molecule chain extenders (such as diamines or diols), which endow them with good mechanical strength [4,5], whereas the soft segments (SS) are formed by oligomeric diols and provide them flexibility and elastic behavior [6,7,8]. Therefore, the material properties can be customized by controlling the ratio of soft and hard segments and structural morphologies [9,10,11], which enable their unique performance, such as excellent wear resistance, high tensile strength, good chemical resistance, and machinability [12,13]. In recent years, TPU play an increasingly important role in many industrial fields, especially in the extensive applications of replacing traditional thermoset elastomers.

In the tire industry, TPU materials are the desirable material to replace rubber materials for the manufacture of high-performance tires, because of good elasticity and thermoplastic processing [14,15]. Currently, the number of waste tires has substantially increased with rapidly growing automobile and transportation development [15,16,17]. The accumulation of waste tires has caused serious environmental pollution problems globally as the rubber material constituting the main material of tires are not biodegradable [18,19,20]. However, compared with rubber materials, TPU materials can be recycled and reused, which will greatly reduce the environmental pollution of waste tires [21,22,23]. Meanwhile, due to the simple processing technology of TPU materials, low rolling resistance, excellent wear resistance, and high mechanical properties [1,6], tires made of TPU materials will have the advantages of low energy consumption, large carrying capacity, and long service life [23,24]. In addition, due to the shortcomings of pneumatic tires, such as poor puncture resistance, easy puncture, low safety factor, and short service life during use, the research of non-pneumatic tires has gradually become one of the research hotspots in the tire field [25,26,27]. For non-pneumatic tires, the structure of the tire has a crucial influence on its performance [25,26,28]. Through the special design of the structure of non-pneumatic tires, it can better meet the application under some complex conditions. However, due to the limitation of mold design in traditional processing methods, the structural design of non-pneumatic tires is single, which greatly restricts the development of non-pneumatic tires.

In recent years, the rise of 3D printing technology has provided a strong guarantee for the manufacture of complex structures of non-pneumatic tires. 3D printing technology, also known as additive manufacturing, is a unique manufacturing philosophy [29,30,31]. Compared with traditional processes, 3D printing technology can directly fabricate products with complex structures with the absence of molds, which are difficult to achieve using traditional processes [32,33,34]. The application of 3D printing technology to the manufacture of non-pneumatic tires can not only greatly increase the freedom of non-pneumatic tire structure design, but also quickly verify the feasibility of tire structure design. Currently, 3D printing technologies based on TPU materials mainly include fused deposition modeling (FDM) and selective laser sintering (SLS) [35,36,37]. FDM technology has become the most widely used 3D printing technology because of its simple operation, relatively cheap equipment cost, and wide range of materials [38,39,40]. Therefore, the application of FDM-based 3D printing technology to the research of non-pneumatic tires has important research significance.

The main research purpose of this study was to evaluate the feasibility of using FDM technology to 3D print non-pneumatic tires based on TPU materials, in order to produce non-pneumatic tires with excellent performance. First, the influence of 3D printing process parameters based on FDM technology on the performance of molded products was studied. Then, on this basis, non-pneumatic tires were successfully manufactured through FDM technology, and the performance of non-pneumatic tires was studied.

## 2. Materials and Methods

### 2.1. Material

The TPU filaments under investigation was acquired Aurora Silver Technology Co., Ltd., Shenzhen, China. The Shore A hardness is 95 A, the filament diameter is 1.75 mm, and the printing temperature is 210–220 °C according to the technical data sheet.

### 2.2. Experimental Methods on 3D Printing Process Parameters

**Figure 1:**
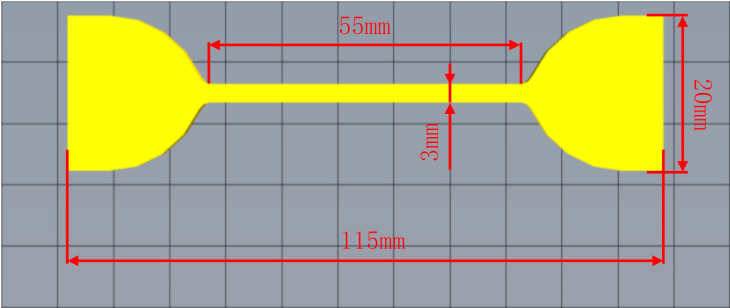
Geometry of dumbbell-shaped tensile specimen.

**Figure 2:**
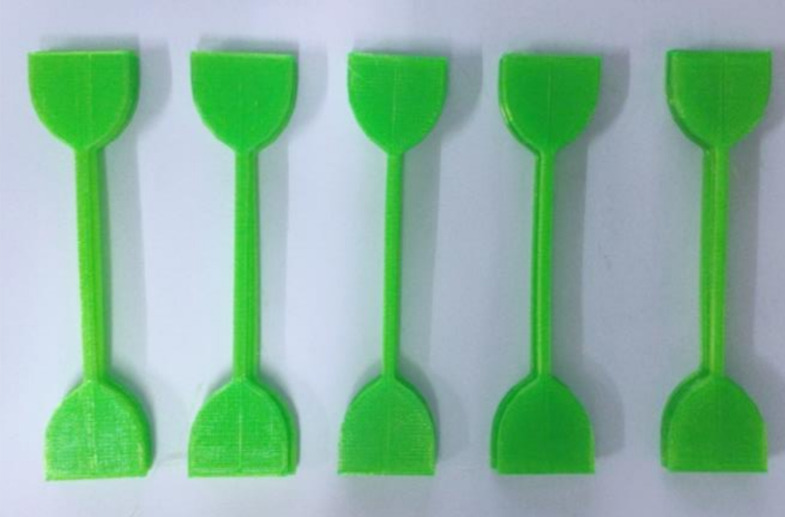
Dumbbell-shaped TPU specimens 3D printed by FDM technology.

To ensure the excellent performance of 3D printed non-pneumatic tires, the relationship between the FDM printing process based on TPU materials and the performance of 3D printed products was studied. The 3D printer used in this experiment is PMAX T15000 (Shenzhen PMAX Investment Development Co., Ltd., Shenzhen, China). In the experiment, we used the dumbbell-shaped tensile specimen shown in Figure 1. While maintaining a single variable, we set different 3D printing temperature or 3D printing fill rate, and then 3D printed the dumbbell-shaped tensile sample. The 3D printed dumbbell-shaped TPU specimens is shown in Figure 2. The specific 3D printing process parameter settings of each scheme are shown in Table 1. Finally, the tensile strength of the 3D printed dumbbell-shaped tensile samples under different printing parameters were tested, respectively, and the effects of 3D printing temperature and 3D printing filling percentage on the performance of 3D printed products were studied according to the test results. 

In addition, to analyze the internal structure of 3D printed samples with different printing parameters, scanning electron microscope (SEM, Hitachi, Tokyo, Japan) was used to observe the cross-sectional morphology of the 3D printed dumbbell-shaped samples.

### 2.3. 3D Printing of Non-Pneumatic Tires

**Figure 3:**
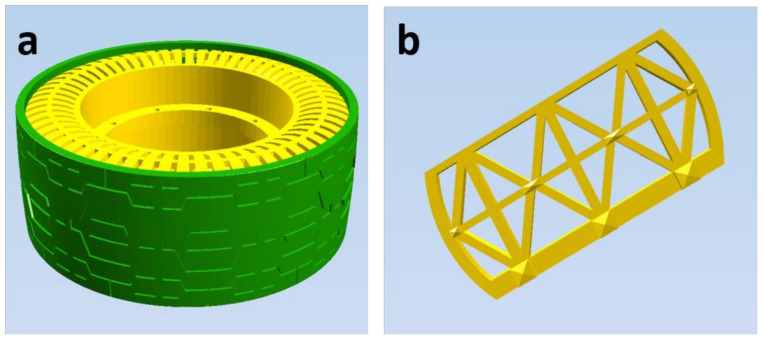
Structure of non-pneumatic tire: (**a**) overall tire structure; and (**b**) spoke structure.

The overall structure of a non-pneumatic tire is shown in Figure 3a. The structure of non-pneumatic tires mainly includes three parts: tread, spokes, and wheel frame. It can be seen in the figure that the tread of the non-pneumatic tire is designed with a relatively complex uneven pattern structure. The design of this structure can increase the wet skid resistance of the non-pneumatic tire. For the tread of non-pneumatic tires during the 3D printing process based on FDM technology, this uneven tread structure on the tread increases the difficulty of 3D printing. To ensure the high-precision 3D printing of the tread structure, the 3D printing speed was reduced and the wall thickness was increased during the 3D printing process.

Figure 3b shows the spoke structure of a non-pneumatic tire. There are 74 spokes evenly distributed inside the non-pneumatic tire, and the performance of the spoke structure is an important guarantee for the overall performance of the non-pneumatic tire. Therefore, a 100% fill percentage is used in the 3D printing molding process of the spoke structure to ensure that the 3D printed spokes have excellent mechanical properties.

The structure at the middle position of the inner wall of the non-pneumatic tire is a wheel frame structure, and some small holes are evenly distributed on the wheel frame structure. The design of the small hole structure is to ensure that the non-pneumatic tire can be installed smoothly with the car rim. Therefore, in the process of 3D printing the wheel frame structure, it is necessary to ensure higher accuracy and integrity. In addition, in the bottom-up 3D printing process of non-pneumatic tires, a suspended structure is formed at the rim. Therefore, support structures need to be added during the 3D printing process of non-pneumatic tires. 

In the experiment, we used FDM technology to 3D printed a non-pneumatic tire based on the above TPU material. Combined with the above research on the 3D printing process of FDM technology, the 3D printing process parameters of non-pneumatic tires are set. The specific 3D printing parameter settings are shown in Table 2. Finally, the performance of non-pneumatic tires was studied.

### 2.4. Measurement and Characterizations

Rheological behavior of the TPU material were measured using capillary rheometer RH 2000 (Malvern instruments limited, Malvern, UK) and rotational rheometer HAAKE-Mars III (Thermo Fisher Scientific (China) Co., Ltd., Shanghai, China) at the melt temperature of 175–230 °C. A STARe system TGA apparatus (Mettler-Toledo International Inc., Greifensee, Switzerland) was applied to test the thermal stability of the TPU material. The sample weighing 10 mg was heated from 25 to 800 °C at a heating rate of 10 °C·min^−1^ under a nitrogen flow. Thermomechanical analysis (TMA) was performed on thermomechanical analyzer (Shimadzu TMA-50, Kyoto, Japan). The sample was heated from 30 to 260 °C at a heating rate of 5 °C·min^−1^ under loading a constant external force of 1 N, and the critical temperature when the thickness of the sample decreases suddenly was defined as the softening temperature. Biaxial tensile test was carried on the biaxial tensile tester (BJDL-W100KN, Jinan Bojian Testing Technology Co., Ltd., Jinan, China). Under the given 25% and 50% strain conditions, the samples were subjected to a cyclic loading–unloading test. Under each given strain condition, the loading–unloading process was repeated 5 times. Five samples were tested and the middle value was taken. The dynamic mechanical property was investigated on a dynamic mechanical analyzer (Rheometric Scientific Co., Limonest, France) with a tension mode at 10 Hz and 3 °C/min from −80 to 100 °C. The Akron abrasion tester (MINGZHU MZ-4061, Mingzhu Testing Machinery Co., Ltd., Yangzhou, China) was used to measure the wear resistance of TPU materials. The abrasion resistance was evaluated based on the volume loss of the sample after a specified number of revolutions (the whole trip was 1.61 km, 3394 revolutions) of the abrasive wheel. The flexural behavior of the TPU material was evaluated according to ISO132 using dynamic fatigue testing machine (GT-7088-D, GOTECH Testing Machines Inc.). Six samples were tested and averaged. Tensile property of the 3D-printed dumbbell-shaped samples was measured according to ASTM D638 using an UN-7001-LAS electrical tensile tester (GOTECH Testing Machines Inc., Qingdao, China) at a rate of 500 mm/min at room temperature. The dimension of the dumbbell-shaped sample for testing was 20 mm in length, 3 mm in width, and 2 mm in thickness. For each measurement, five samples were tested and the average was taken. Scanning electron microscope (SEM) was adopted from Hitachi S-4800 (Japan) to observe the cross-sectional structure of the 3D printed samples. The three-dimensional stiffness of the 3D printed non-pneumatic tire was tested on the flat tire dynamic characteristics test bench (JLU-III, Jilin University, Changchun, China). When a non-pneumatic tire is running, apply a certain load in one direction (Radial, lateral, longitudinal) to test its stiffness in the corresponding direction. The industrial-grade 3D printing printer PMAX T15000 (Shenzhen PMAX Investment Development Co., Ltd., Shenzhen, China) was used to manufacture dumbbell-shaped tensile samples and non-pneumatic tires. The nozzle diameter of the 3D printer is 0.4 mm, and the molding size is 1200 mm × 1200 mm × 1200 mm.

## 3. Results and Discussion

### 3.1. Interaction Between TPU Melt Rheology and Printing Process

**Figure 4:**
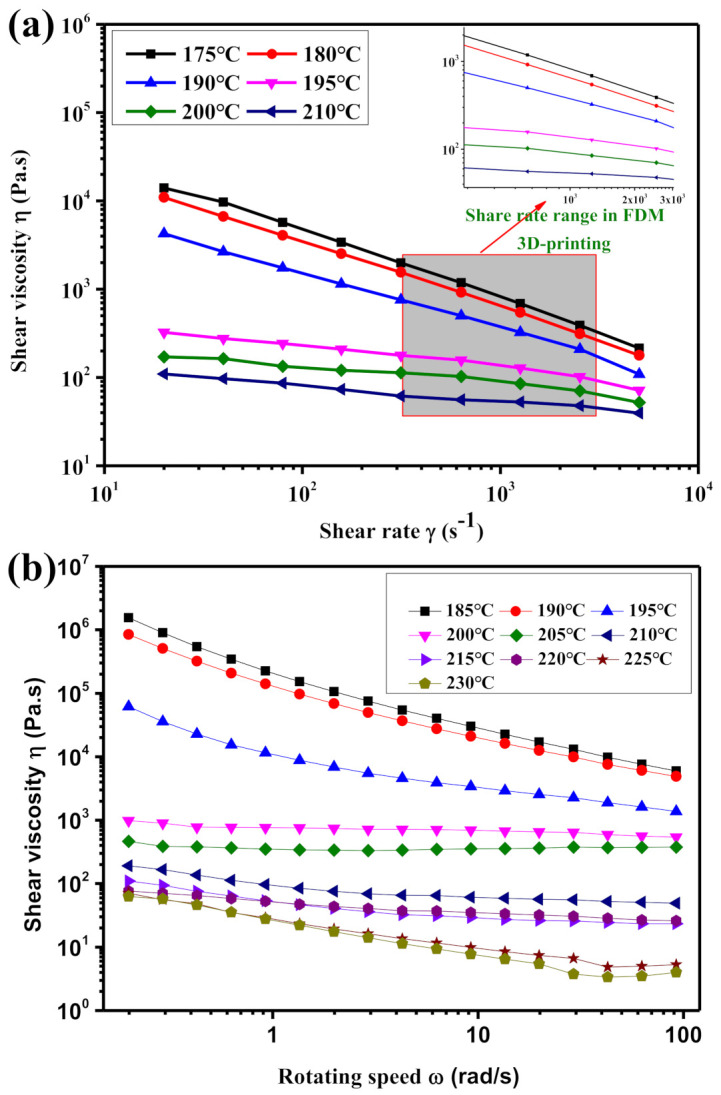
Shear viscosity of TPU melt: (**a**) capillary rheometer; and (**b**) rotary rheometer.

During the 3D printing process of FDM technology, the solid filament material was heated at the nozzle to reach a molten state, and then extruded from the nozzle and deposited on the printing platform. In this process, the fluidity of the molten filaments has a vital influence on the stability of the 3D printing process. Therefore, to study the flow behavior of molten filaments, capillary rheometer, and rotational rheometer were used to test the relationship between the shear rate and shear viscosity of TPU materials at different temperatures. The results are shown in Figure 4.

For FDM technology, the filament feeding method is to use two driving wheels to provide a traction to the filamentous material, and then the filament is transported through a catheter to the nozzle of the 3D printer and heated and melted at the nozzle. However, because the TPU material itself is a soft material. In addition, due to the heat transfer effect generated at the hot nozzle, the TPU filaments in the throat are further softened. It is well known that filaments with less rigidity cannot effectively transmit traction. In addition, when the molten filament is extruded from the nozzle, the higher is the viscosity, the greater is the extrusion force required. Therefore, to ensure the printing stability of the TPU filament in the 3D printing process based on the FDM technology, the viscosity of the molten filament at the nozzle should be appropriately controlled. Figure 4 shows that, at the same test temperature, as the shear rate increases, the shear viscosity of the TPU melt gradually decreases. At the same shear rate, as the test temperature increases, the shear viscosity of the TPU melt gradually decreases. However, since the nozzle diameter of the FDM printer is 0.4 mm, when the 3D printing speed is 10–100 mm/s, the shear rate of the molten TPU during nozzle extrusion is about 500–3000 s^−1^. Figure 4a shows that, in the shear rate range of 500–3000 s^−1^, when the melt temperature is 175, 180, and 190 °C, the shear viscosity of the melt TPU is larger. This may cause the molten TPU filaments to be unable to be stably extruded from the nozzle for a long time. When the melt temperature reaches 195 °C, the shear viscosity drops rapidly. This may be because the TPU material is fully melted, and the melt viscosity is relatively suitable at this time. Therefore, to ensure the stability of the TPU filament 3D printing process, the 3D printing temperature should be controlled above 195 °C.

### 3.2. Research on the Thermal Properties of TPU Materials

**Figure 5:**
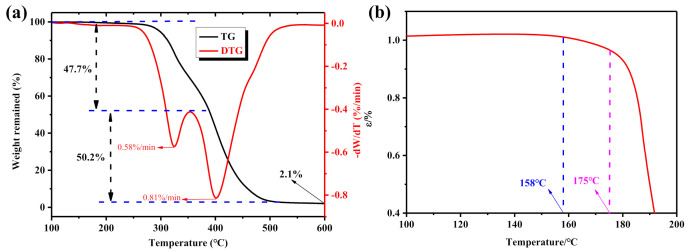
Thermal properties of TPU material: (**a**) TGA and DTG curves; and (**b**) TMA curve.

According to the rheological analysis of TPU materials, to ensure the stability of the melt extrusion of TPU filament from the hot nozzle during the 3D printing process, the 3D printing temperature should be appropriately increased to reduce the shear viscosity of the melt filaments. However, when the 3D printing temperature is too high, the TPU material is prone to decomposition, resulting in a decrease in the performance of the 3D printed product. Therefore, thermogravimetric analysis was used to study the thermal stability of TPU materials, and the results are shown in Figure 5a. As shown in Figure 5a, when the temperature reaches about 250 °C, the thermogravimetric curve of the TPU material begins to drop significantly, which indicates that the TPU material has begun to decompose at 250 °C. Therefore, the 3D printing temperature for TPU filaments should be controlled below 250 °C.

In addition, for non-pneumatic tires made of TPU material, when the tire is driving, the tire and the ground rub against each other to generate heat, which will cause the TPU material to become soft and affect the driving performance of the tire. Therefore, to ensure the normal driving of TPU tires, the softening temperature of TPU materials was studied by thermomechanical analysis (TMA), and the results are shown in Figure 5b. As shown in Figure 5b, when the temperature reaches 158 °C, the TPU material starts to soften slowly. With the further increase in temperature, when the temperature reaches 175 °C, the TMA curve begins to drop sharply, which indicates that the TPU material has completely softened, and the load of the TPU non-pneumatic tire will decrease significantly at this time. Therefore, when using TPU materials to manufacture non-pneumatic tires, the effect of the softening temperature of TPU materials on the running performance of the tire should be considered. Moreover, according to the previous analysis in this article, during the 3D printing process, the heat transfer effect at the hot nozzle will soften the TPU filaments, thereby affecting the melt extrusion of the TPU filaments at the nozzle. As for the heat transfer effect at the hot nozzle, when the nozzle temperature is higher, the heat transfer effect is more obvious, which ultimately leads to the faster the temperature increase rate of the TPU filament at the catheter. Therefore, in the 3D printing process of TPU filaments, the 3D printing temperature should be avoided too high to reduce the influence of the heat transfer effect at the hot nozzle on the stability of the printing process.

### 3.3. Research on Dynamic Mechanical Properties of TPU Materials

**Figure 6:**
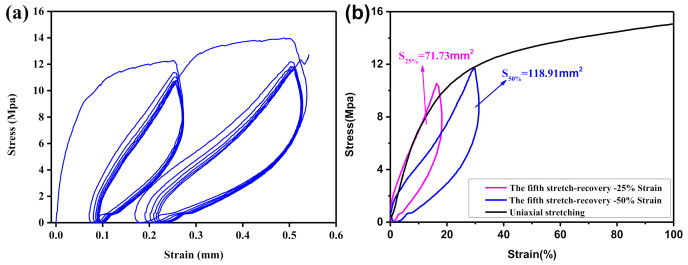
(**a**) Tensile–recovery curve of TPU material under cyclic loading–unloading; and (**b**) comparison of the fifth stretch recovery and uniaxial stretch curves of TPU material.

When the tire is driving, the stress acting on the tire material changes with time. Therefore, to better study the mechanical properties of tire materials, the isobiaxial tensile test was used to study the relationship between the stress and strain of the TPU material in the cyclic stretching–recovery process. The test result is shown in Figure 6. During the cyclic loading–unloading process of the TPU material, under the same strain conditions, the tensile curve and the recovery curve do not completely overlap, resulting in hysteresis. This is mainly because, during the loading–unloading process, the movement of the TPU macromolecular segment is hindered by internal frictional resistance, resulting in strains that cannot keep up with the changes in stress. In a cyclic loading–unloading process, the area enclosed by the stretching curve and recovery curve represents the energy loss caused by internal frictional resistance in the movement process of macromolecular chain segments. Analogous to the movement process of non-pneumatic tires, the energy loss represented by the area of the hysteresis ring of TPU material reflects the energy consumption of non-pneumatic tires during the movement.

To reflect the energy consumption of TPU non-pneumatic tires more truly during driving, the fifth cyclic stretch–recovery curves under each given strain condition was intercepted and compared with the uniaxial stretch curve. The result is shown in Figure 6b. The highest points of the uniaxial stretching and cyclic stretching–recovery curves basically coincide, which shows the accuracy of the isobiaxial stretching test results. Moreover, as the given strain increases, the area of the hysteresis loop enclosed by the fifth cyclic stretch–recovery curve gradually increases. When under the given 25% strain condition, the area of the hysteresis ring is 71.73 mm^2^, and, under the given 50% strain condition, the area of the hysteresis ring is 118.91 mm^2^. This shows that non-pneumatic tires made by TPU materials can reduce the energy consumption of the tires by appropriately reducing the load during driving.

**Figure 7:**
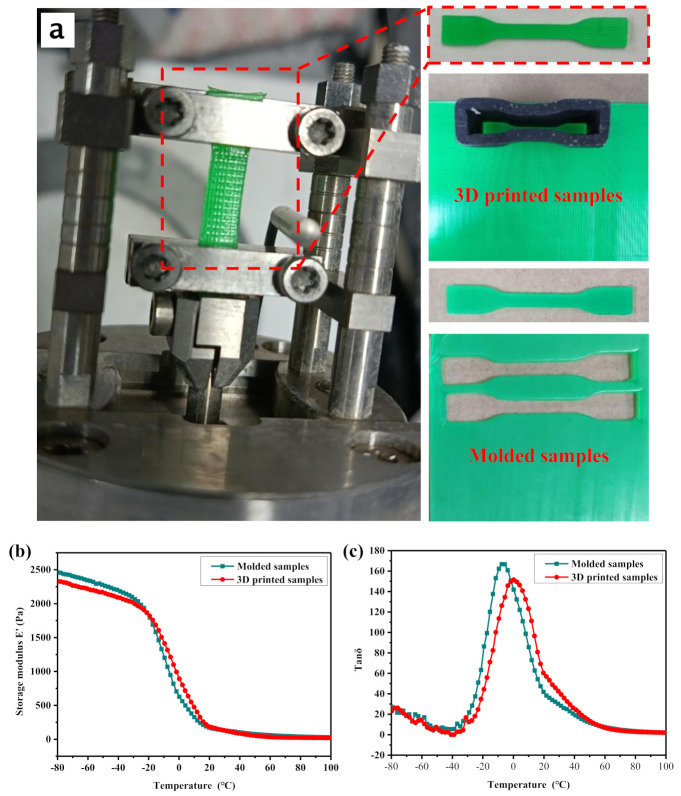
DMA curves of TPU material: (**a**) DAM test process; (**b**) storage modulus (E′) versus temperature; and (**c**) Tanδ versus temperature.

Storage modulus and Tanδ curves of the molded sample and 3D printed sample prepared with TPU materials as a function of temperature are shown in Figure 7b,c, respectively. The storage modulus E’ represents the rigidity of the material. The greater is the storage modulus E’, the greater is the rigidity of the material, which also means the greater is the resistance of the material to deformation. For tire materials, the response relationship between its storage modulus and temperature has a greater impact on tire performance. When the tire is driving, the friction between the tire and the ground will cause the temperature of the tire material to rise. As the temperature increases, the smaller is the change trend of the storage modulus of the tire material, the better is the retention rate of the tires performance. Figure 7b shows that, as the temperature increases, the storage modulus of the molded product and the 3D printed product have basically the same changing trend, and the two curves basically coincide. This indicates that the difference in dynamic mechanical properties between 3D printed samples and molded samples is relatively small, which also provides a certain feasibility for 3D printing and molding of non-pneumatic tires.

In addition, Figure 7c shows that compared with the molded sample, as the temperature increases, the peak value of Tanδ of the 3D printed sample decreases, and the temperature corresponding to the peak value decreases. The value of Tanδ represents the internal friction of the material under dynamic load. The larger is the value of Tanδ, the greater is the internal friction of the material under dynamic load. The temperature corresponding to the peak of Tanδ represents the glass transition temperature of the material. This indicates that the internal friction of the 3D printed sample under dynamic load is reduced, and the glass transition temperature of the material becomes larger. This may be due to the higher orientation of the TPU material of the 3D printed samples. When the molten TPU filaments are extruded from the hot nozzle and deposited on the printing platform, the movement of the printer nozzle causes the melt-extruded filaments to produce tensile orientation during the deposition process. The increase in the degree of orientation leads to the weakening of the mobility of the segments in the TPU macromolecule, so it has lower internal friction and glass transition temperature under the action of dynamic load.

**Figure 8:**
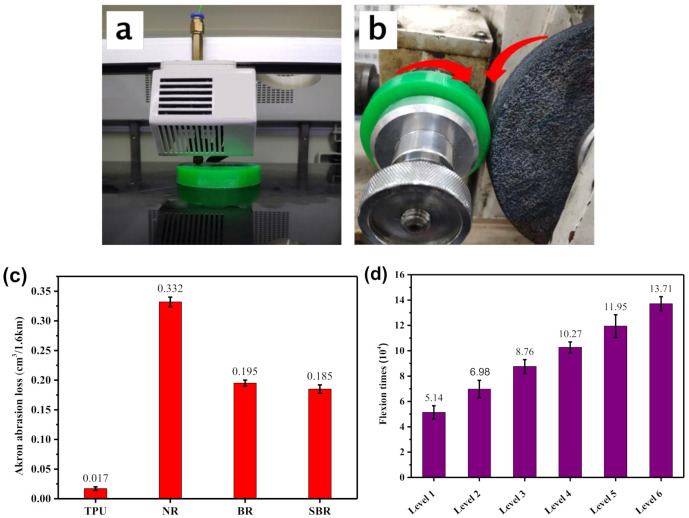
3D printing process of Akron abrasion test samples (**a**); test process of Akron abrasion test samples (**b**); (**c**) Akron abrasion; and (**d**) flex cracking.

For tire materials, its wear resistance is a crucial factor affecting tire performance. In this experiment, non-pneumatic tires were manufactured using FDM-based technology. Therefore, to better study the abrasion resistance of non-pneumatic tires made by FDM technology, the Akron abrasion test was performed on the TPU material, and experimental samples were prepared using 3D printing technology. At the same time, the test results of TPU materials were compared with those of natural rubber (NR), butadiene rubber (BR), and styrene butadiene rubber (SBR). The results are shown in Figure 8a–c. Figure 8c shows that the Akron abrasion loss of TPU material is significantly lower than that of other rubber materials, which shows that the wear resistance of TPU material is better than that of other rubber materials. In addition, to study the fatigue resistance of the TPU material, a flexural resistance test was performed on the TPU material, and the results are shown in Figure 8d. The number of first-level flexions of TPU material reached 51.4 thousand, and the number of sixth-level flexions reached 137.1 thousand. This shows that TPU materials have relatively excellent fatigue resistance.

### 3.4. Research on 3D Printing Process Parameters

Table 3 shows the tensile test results of the 3D printed dumbbell-shaped tensile samples when the 3D printing parameters are set as shown in Table 1. For each measurement, five samples were tested and the average was taken. Combined with the test results in Table 2, the effects of 3D printing temperature and 3D printing filling percentage on the performance of 3D printing products were studied.

**Figure 9:**
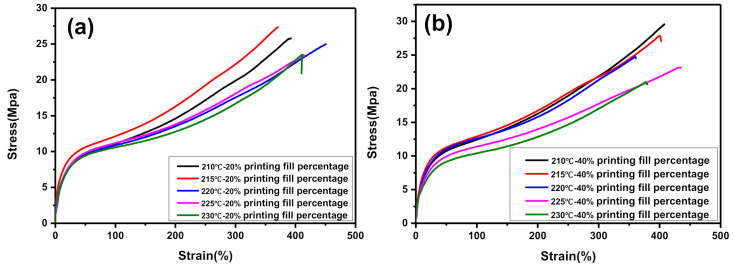

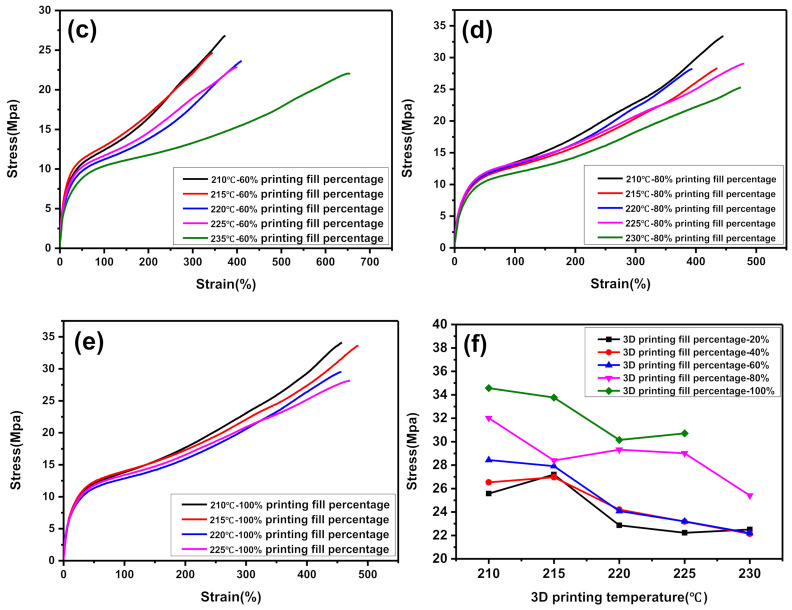
The relationship between 3D printing temperature and the tensile strength of 3D printed dumbbell-shaped tensile samples: (**a**) 20% printing fill percentage; (**b**) 40% printing fill percentage; (**c**) 60% printing fill percentage; (**d**) 80% printing fill percentage; (**e**) 100% printing fill percentage; and (**f**) 3D printing temperature.

Figure 9a–e shows the stress–strain curves of 3D printed dumbbell-shaped tensile samples at different 3D printing temperatures. Figure 9a–e shows that, under the same 3D printing filling rate, with the change of 3D printing temperature, the change trend of the tensile properties of the 3D printing samples shows some differences. When the 3D printing filling percentage is 20% and 40%, the tensile strength of the printed sample with 3D printing temperature of 215 °C is the largest. When the 3D printing filling percentage is 60%, 80%, and 100%, the tensile strength of the printed sample with 3D printing temperature of 210 °C is the highest. To better study the effect of 3D printing temperature on 3D printed products, the tensile strength of 3D printed dumbbell-shaped tensile samples at different 3D printing temperatures was compared. The results are shown in Figure 9f. When the 3D printing temperature is 210 °C and the 3D printing filling percentage is 100%, the tensile strength of the 3D printing sample is the largest. This indicates that selecting 210 °C as the 3D printing temperature for non-pneumatic tires may be more beneficial to the performance of 3D printed non-pneumatic tires.

**Figure 10:**
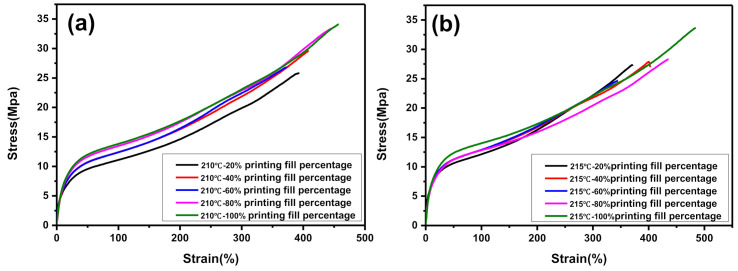

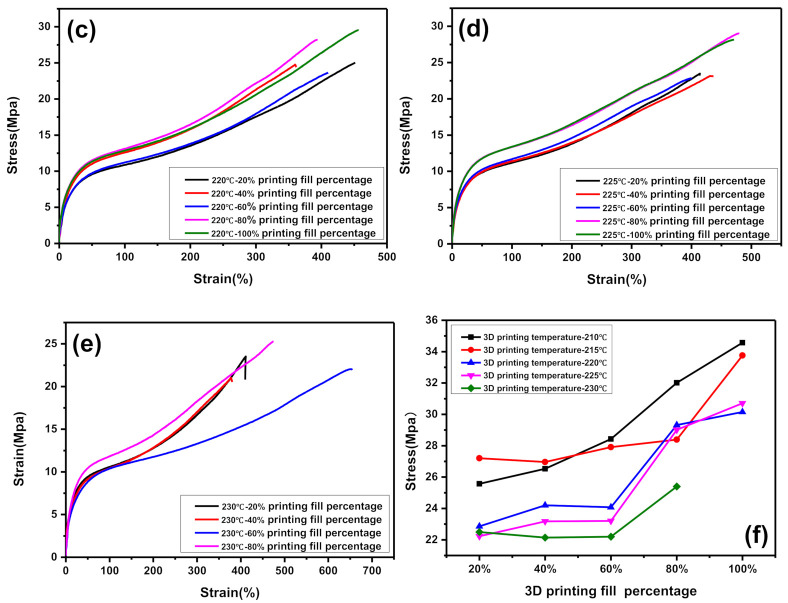
The relationship between 3D printed fill percentage and the tensile strength of 3D printed dumbbell-shaped tensile samples: (**a**) 3D printing temperature of 210 °C; (**b**) 3D printing temperature of 215 °C; (**c**) 3D printing temperature of 220 °C; (**d**) 3D printing temperature of 225 °C; (**e**) 3D printing temperature of 230 °C; and (**f**) 3D printing fill percentage.

For the study of 3D printing fill percentage, the data processing method is the same as the study of 3D printing temperature. As shown in Figure 10f, as the percentage of 3D printing fill increases, the tensile strength of the 3D printed dumbbell-shaped tensile sample gradually increases. In addition, with different 3D printing fill percentages, the tensile strength of the 3D printed dumbbell-shaped tensile samples varies greatly. When the 3D printing temperature is maintained at 215, 220, and 225 °C, the tensile strength of the 3D printed dumbbell-shaped tensile sample differs by about 5 MPa between the 20% 3D printing fill percentage and the 100% 3D printing fill percentage.

**Figure 11:**
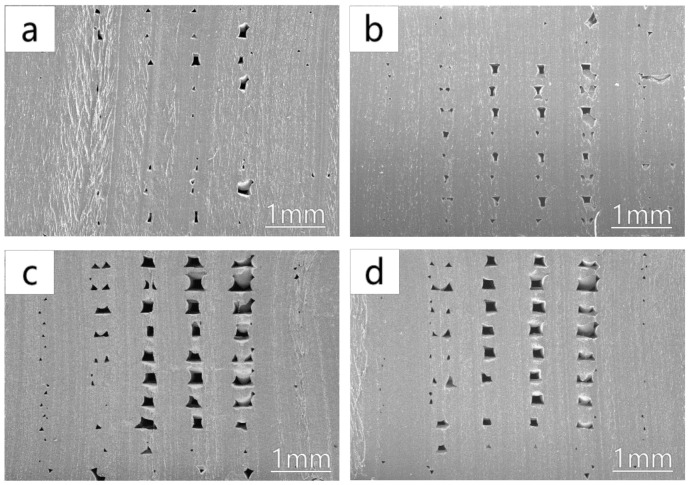
SEM image of the cross-section of each 3D printed dumbbell-shaped tensile sample at different 3D printing temperatures when the 3D printing filling percentage is 100%: (**a**) 3D printing temperature of 210 °C; (**b**) 3D printing temperature of 215 °C; (**c**) 3D printing temperature of 220 °C; and (**d**) 3D printing temperature of 225 °C.

It can be seen from the above analysis that when the 3D printing fill percentage is 100% and the 3D printing temperature is 210 °C, the performance of the 3D printed dumbbell-shaped tensile sample is the best. Figure 11 shows the scanning electron microscope observation results of the cross-section of the 3D printed dumbbell-shaped tensile samples. When the 3D printing fill percentage is 100%, as the 3D printing temperature increases, the internal void ratio of the 3D printed dumbbell-shaped tensile sample gradually increases. When the 3D printing temperature is 210 °C, the internal voids of the 3D printed dumbbell-shaped tensile specimen are the smallest. As is known, the internal defects of products have a great impact on the mechanical properties of products. The more defects there are inside the product, the worse the mechanical properties of the product are. During the tensile test, due to the stress concentration effect, the stress at the defect increases, causing the product to start to break from the stress concentration point, and quickly leading to the complete failure of the product. Therefore, to reduce the internal defects of the 3D printed non-pneumatic tires, thereby ensuring the overall performance of the tires, 210 °C was selected as the 3D printing temperature of the non-pneumatic tires.

In addition, when the 3D printing fill percentage is 100%, it means that the interior of the 3D printed product should be a solid structure. However, according to the scanning electron microscopy images of the cross-sections of 3D printed dumbbell-shaped tensile samples with 100% filling percentage at different 3D printing temperatures, it can be seen that there are more or fewer void structures inside the 3D printed products. It is believed that the further decrease of voids can improve the performance of 3D printed products using FDM technology.

### 3.5. Research on the Performance of 3D Printed Non-Pneumatic Tires

**Figure 12:**
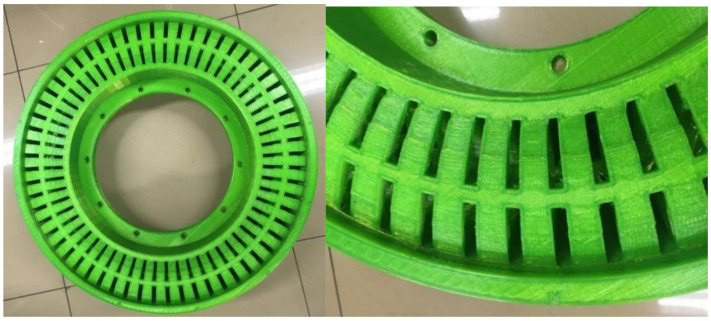
3D printed non-pneumatic tire based on TPU material.

Figure 12 shows a 3D printed non-pneumatic tire based on FDM technology using TPU material. It can be seen in the figures that the final 3D printed non-pneumatic tire has a smooth surface and high overall structure accuracy. At the same time, the addition of the supporting structure ensures the 3D printing accuracy of the wheel frame structure. The small holes on the wheel frame are clearly visible and uniform in size. The small holes on the wheel frame are clearly visible and the size is uniform, which greatly ensures the installation of the rim structure afterwards.

**Figure 13:**
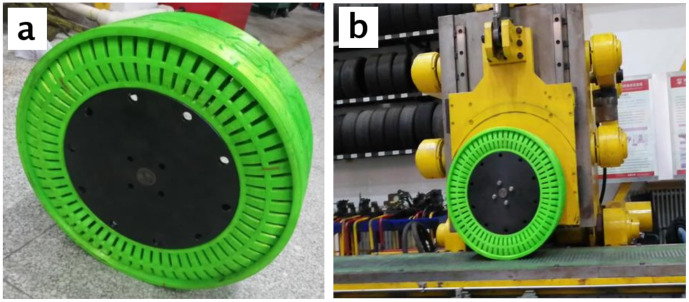
(**a**) Non-pneumatic tire with rim structure installed; and (**b**) performance test chart of non-pneumatic tires.

Figure 13a shows the overall structure of the non-pneumatic tire after the rim structure is installed. After the 3D printing of the non-pneumatic tire is completed, it needs to be installed on the car through the rim structure to ensure its normal driving. As shown in Figure 13b, the performance test process of the non-pneumatic tire with the rim structure installed. In this experiment, we tested the three-dimensional stiffness of 3D printed non-pneumatic tires and compared the test results with computer simulation values.

**Figure 14:**
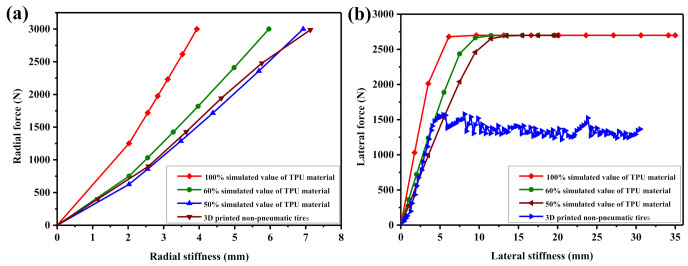

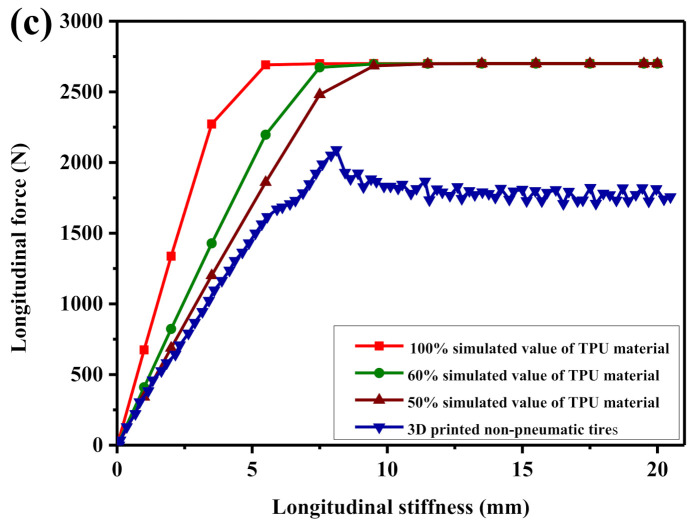
Comparison of the actual three-dimensional stiffness curve of the 3D printed non-pneumatic tire and the simulated curves: (**a**) radial stiffness curves; (**b**) lateral stiffness curves; and (**c**) longitudinal stiffness curve.

Figure 14 shows the three-dimensional stiffness curve of the non-pneumatic tire. The simulated values of 100%, 60%, and 50% of the TPU material in the figure represent the simulated stiffness values of the non-pneumatic tires obtained by using 100%, 60%, and 50% of the performance of the TPU material. In addition, the slope values of the curves in the figures are the stiffness values of the non-pneumatic tires. Figure 14 shows that the slope of the actual three-dimensional stiffness curve of the 3D printed non-pneumatic tire is quite different from the slope of the simulation curve obtained by 100% of the performance of the TPU material. However, as the percentage of TPU material performance decreases gradually, the slope of the actual three-dimensional stiffness curve of the 3D printed non-pneumatic tire gradually matches the slope of the simulation curve.

To better study the experimental and simulation results of the three-dimensional stiffness of 3D printed non-pneumatic tires, we conducted a comparative analysis of the slopes of the curves in Figure 14. The results are shown in Table 4. Table 4 shows that, under the load of 3000 N, the actual three-dimensional stiffness values of the 3D printed non-pneumatic tires are as follows: the radial stiffness value is 462 N/mm, the lateral stiffness value is 344.4 N/mm, and the longitudinal stiffness value is 306.1 N/mm. Comparing the actual three-dimensional stiffness values of 3D printed non-pneumatic tires, the radial stiffness is the largest, which will help improve the handling stability of the car.

Table 4 also shows that, when 100% of the performance of TPU material is used for the three-dimensional stiffness simulation, the simulation results of non-pneumatic tires are as follows: the radial stiffness value is 762.1 N/mm, the lateral stiffness value is 437.5 N/mm, and the longitudinal stiffness value is 489.2 N/mm. At this time, the actual lateral stiffness of the non-pneumatic tire is only 78.7% of the simulated value, and the actual lateral and longitudinal stiffnesses can only reach about 60.6% of the simulated value. This shows that, when using FDM technology for 3D printing of non-pneumatic tires, due to some defects in the molded products, the performance loss of the final non-pneumatic tires is relatively large.

When 60% of the performance of TPU material is used for the three-dimensional stiffness simulation, the actual lateral stiffness of the non-pneumatic tires reaches 107% of the simulated value. At this time, the actual lateral stiffness is not much different from the simulation result. However, the actual lateral stiffness and actual longitudinal stiffness reach about 80% of the simulated value. When 50% of the performance of TPU material is used for the three-dimensional stiffness simulation, the actual lateral stiffness and actual longitudinal stiffness of the non-pneumatic tires reach 90% of the simulation results. This shows that, by reducing the material performance and simulation, it can be guaranteed that the actual three-dimensional stiffness of the non-pneumatic tire based on FDM technology 3D printing is consistent with the simulation value, so that the performance of the non-pneumatic tire can be better predicted.

## 4. Conclusions

In this study, we used TPU materials to study the effect of 3D printing technology based on FDM on the performance of non-pneumatic tires. The analysis of the rheological properties of TPU materials shows that, as the shear rate increases, the shear viscosity gradually decreases. Within the shear rate range of FDM processing, to ensure the stability of the 3D printing process, the 3D printing temperature needs to reach at least 195 °C. The wear resistance comparison results show that the wear resistance of TPU materials is much better than natural rubber (NR), butadiene rubber (BR), and styrene butadiene rubber (SBR). The research on the 3D printing process parameters of TPU material shows that, with the increase of 3D printing filling percentage, the performance of 3D printing samples gradually improves. When the 3D printing temperature is 210 °C and the 3D printing filling rate is 100%, the performance of the 3D printing sample is optimal.In addition, combined with the exploration of the 3D printing process of TPU materials, non-pneumatic tires were successfully manufactured using FDM technology, and the three-dimensional stiffness of 3D printed non-pneumatic tires was studied. By reducing the performance of the material used for the simulation, the actual three-dimensional stiffness of the 3D printed non-pneumatic tire can be made consistent with the simulation result.

## Figures and Tables

**Table 1:** 3D printing process parameter settings in each scheme.

Program	3D Printing Temperature (°C)	3D Fill Percentage (%)	Other 3D Printing Parameter Settings
1#	210	20%	Constant
2#	210	40%	Constant
3#	210	60%	Constant
4#	210	80%	Constant
5#	210	100%	Constant
6#	215	20%	Constant
7#	215	40%	Constant
8#	215	60%	Constant
9#	215	80%	Constant
10#	215	100%	Constant
11#	220	20%	Constant
12#	220	40%	Constant
13#	220	60%	Constant
14#	220	80%	Constant
15#	220	100%	Constant
16#	225	20%	Constant
17#	225	40%	Constant
18#	225	60%	Constant
19#	225	80%	Constant
20#	225	100%	Constant
21#	230	20%	Constant
22#	230	40%	Constant
23#	230	60%	Constant
24#	230	80%	Constant

**Table 2:** 3D printing parameter values of non-pneumatic tires.

Parameters	Values
Nozzle aperture (mm)	0.4
Layer thickness (mm)	0.3
Wall thickness (mm)	1.6
Top/bottom thickness	1.6
Fill percentage (%)	100
Intermediate printing speed(mm/s)	30
Top/bottom printing speed (mm/s)	20
3D printing temperature (°C)	210
Support type	All
Adhesion platform	Raft

**Table 3:** Mechanical properties of 3D printed dumbbell-shaped tensile samples.

Program	Tensile Strength (MPa)	Elongation at Break (%)	100% Fixed Stretch (MPa)	300% Fixed Stretch (MPa)
1#	25.8 ± 0.7	393.7 ± 28	11.1 ± 0.5	19.9 ± 0.4
2#	29.6 ± 1.0	408.8 ± 26	12.4 ± 0.6	21.9 ± 0.8
3#	26.8 ± 0.5	373.4 ± 30	12.4 ± 0.6	22.4 ± 0.9
4#	33.4 ± 0.4	445.4 ± 25	13.5 ± 0.9	22.9 ± 0.5
5#	34.1 ± 0.8	457.4 ± 23	13.8 ± 0.9	23.1 ± 1.1
6#	27.4 ± 1.2	371.7 ± 29	12.2 ± 1.1	22.2 ± 0.9
7#	27.9 ± 0.5	403.0 ± 22	12.9 ± 0.6	21.8 ± 0.9
8#	24.7 ± 0.8	344.9 ± 28	12.9 ± 0.9	22.1 ± 0.6
9#	28.3 ± 0.7	435.7 ± 32	12.8 ± 0.4	20.4 ± 0.7
10#	33.7 ± 0.6	484.6 ± 18	14.0 ± 0.5	22.1 ± 0.8
11#	25.0 ± 0.4	451.5 ± 22	10.9 ± 0.3	17.5 ± 0.7
12#	24.8 ± 0.6	361.5 ± 24	12.6 ± 0.5	21.3 ± 0.3
13#	23.7 ± 0.7	410.3 ± 26	11.2 ± 0.9	18.0 ± 0.6
14#	28.2 ± 1.1	394.2 ± 27	13.1 ± 0.9	22.2 ± 0.8
15#	29.6 ± 0.5	456.8 ± 26	12.9 ± 0.8	20.6 ± 1.1
16#	23.5 ± 0.3	414.1 ± 23	11.2 ± 0.9	18.1 ± 0.6
17#	23.2 ± 0.8	436.6 ± 30	11.4 ± 0.4	17.8 ± 0.9
18#	22.9 ± 0.9	400.3 ± 29	11.7 ± 1.0	19.0 ± 0.7
19#	29.0 ± 1.0	479.9 ± 24	13.4 ± 0.9	20.8 ± 0.4
20#	28.1 ± 0.4	470.0 ± 26	13.4 ± 0.4	20.9 ± 0.4
21#	23.5 ± 0.6	411.5 ± 29	10.6 ± 0.8	16.7 ± 0.5
22#	21.0 ± 0.4	380.0 ± 24	10.4 ± 0.6	17.0 ± 0.7
23#	22.0 ± 0.9	654.9 ± 22	10.4 ± 0.4	13.3 ± 0.9
24#	25.3 ± 0.6	474.7 ± 26	11.8 ± 0.4	18.3 ± 0.4

**Table 4:** Comparison of actual three-dimensional stiffness values of non-pneumatic tires with simulated three-dimensional stiffness values.

Direction	Load	Experimental Values (N/mm)	100% Simulation of TPU Materials		60% Simulation of TPU Materials		50% Simulation of TPU Materials	
			Simulation Values (N/mm)	Comparative Experiment Value (%)	Simulation Values (N/mm)	Comparative Experiment Value (%)	Simulation Values (N/mm)	Comparative Experiment Value (%)
Radial	/	462	762.1	60.6%	560.8	80.3%	473.6	97.5%
Lateral	3000 N	344.4	437.5	78.7%	320.1	107%	268.2	128%
Portrait	3000 N	306.1	489.2	62.6%	396.8	77.1%	334.8	91.4%
